# Pharmacokinetics, adverse effects and effects on thermal nociception following administration of three doses of codeine to horses

**DOI:** 10.1186/s12917-022-03299-0

**Published:** 2022-05-25

**Authors:** Heather K. Knych, Kristen Stucker, Sophie R. Gretler, Philip H. Kass, Daniel S. McKemie

**Affiliations:** 1 K.L. Maddy Equine Analytical Pharmacology Laboratory, School of Veterinary Medicine, University of California, Davis, CA 95616 USA; 2 Department of Molecular Biosciences, School of Veterinary Medicine, University of California, Davis, CA USA; 3 Department of Population Health and Reproduction, School of Veterinary Medicine, University of California, Davis, CA USA

**Keywords:** Horse, Opioid, Codeine, Pharmacokinetics, Thermal, Analgesia, Metabolism

## Abstract

**Background:**

In humans, codeine is a commonly prescribed analgesic that produces its therapeutic effect largely through metabolism to morphine. In some species, analgesic effects of morphine have also been attributed to the morphine-6-glucuronide (M6G) metabolite. Although an effective analgesic, administration of morphine to horses produces dose-dependent neuroexcitation at therapeutic doses. Oral administration of codeine at a dose of 0.6 mg/kg has been shown to generate morphine and M6G concentrations comparable to that observed following administration of clinically effective doses of morphine, without the concomitant adverse effects observed with morphine administration. Based on these results, it was hypothesized that codeine administration would provide effective analgesia with decreased adverse excitatory effects compared to morphine. Seven horses received a single oral dose of saline or 0.3, 0.6 or 1.2 mg/kg codeine or 0.2 mg/kg morphine IV (positive control) in a randomized balanced 5-way cross-over design. Blood samples were collected up to 72 hours post administration, codeine, codeine 6-glucuronide, norcodeine morphine, morphine 3-glucuronide and M6G concentrations determined by liquid chromatography- mass spectrometry and pharmacokinetic analysis performed. Pre- and post-drug related behavior, locomotor activity, heart rate and gastrointestinal borborygmi were recorded. Response to noxious stimuli was evaluated by determining thermal threshold latency.

**Results:**

Morphine concentrations were highest in the morphine dose group at all times post administration, however, M6G concentrations were significantly higher in all the codeine dose groups compared to the morphine group starting at 1 hour post drug administration and up to 72-hours in the 1.2 mg/kg group. With the exception of one horse that exhibited signs of colic following administration of 0.3 and 0.6 mg/kg, codeine administration was well tolerated. Morphine administration, led to signs of agitation, tremors and excitation. There was not a significant effect on thermal nociception in any of the dose groups studied.

**Conclusions:**

The current study describes the metabolic profile and pharmacokinetics of codeine in horses and provides information that can be utilized in the design of future studies to understand the anti-nociceptive and analgesic effects of opioids in this species with the goal of promoting judicious and safe use of this important class of drugs.

**Supplementary Information:**

The online version contains supplementary material available at 10.1186/s12917-022-03299-0.

## Background

Codeine is an opioid and a naturally occurring alkaloid used for relief of mild to moderate pain, as a cough suppressant and as an anti-diarrheal [1]. Although not as commonly used in veterinary medicine, its use in human medicine, either as a sole agent or in combination with other analgesic and anti-inflammatory medications, is commonplace. Codeine is extensively metabolized with only 2–7% of the administered dose being excreted as the parent compound [2]. In humans, 5 different metabolites have been reported in vivo, including codeine 6-glucuronide (C6G) (81%), norcodeine (2.2%), morphine 3-glucuronide (M3G) (2.1%), morphine 6-glucuronide (M6G) (0.8%) and morphine (0.56%) [3]. The analgesic effects of codeine in humans have been largely attributed to metabolism to morphine, with morphine reportedly ten times more potent than the parent compound [4]. Interestingly, there are a number of published studies in humans and rats that have attributed the analgesic effects of morphine administration, at least in part, to the M6G metabolite [5–8]. There are also reports suggesting that C6G contributes to the antinociceptive effects following codeine administration [9–11].

There are a limited number of published studies describing the disposition and/or pharmacologic effects of codeine in the horse [12, 13]. In a recent study, codeine administration to horses was shown to generate a similar metabolic profile to that observed in other species, including production of morphine and M6G [14]. One notable finding from this study was that morphine and M6G concentrations following oral administration of 0.6 mg/kg codeine were comparable to that observed following administration of the reported analgesic dose of morphine (0.2 mg/kg, intravenous) [15]. While morphine administration at clinically effective doses has been shown to cause neuroexcitation in horses, increases in heart rate and adverse gastrointestinal effects [16–18], oral administration of 0.2 mg/kg of codeine was well tolerated [14].

Although not commonly used as an analgesic in horses, demonstration of the conversion of codeine to morphine and the theorized analgesic metabolite, M6G along with the lack of behavioral excitation support further study of the analgesic properties of this compound in the horse. In the current study, the pharmacokinetics and response to a noxious stimulus following oral administration of 3 doses of codeine was studied and it was hypothesized that administration would provide predictable, time-related blood concentrations of parent drug and active metabolites and increase thermal nociception with minimal adverse effects.

## Results

### Concentration determination and pharmacokinetic analysis

The LC-MS/MS instrument responses for all compounds was linear with correlation coefficients of 0.99 or better. Quality control samples were assayed in replicates (*n* = 6) for determination of precision and accuracy. Accuracy and precision were reported as percent nominal concentration, and percent relative standard deviation, respectively (Supplementary Table 1). The limit of quantitation of the assay was of 0.1 ng/mL for codeine, C6G, norcodeine and M3G and 0.25 ng/mL for morphine and M6G while the limit of detection was approximately 0.05 ng/mL for codeine, C6G, norcodeine and M3G and 0.1 ng/mL for morphine and M6G.

**Fig. 1:**
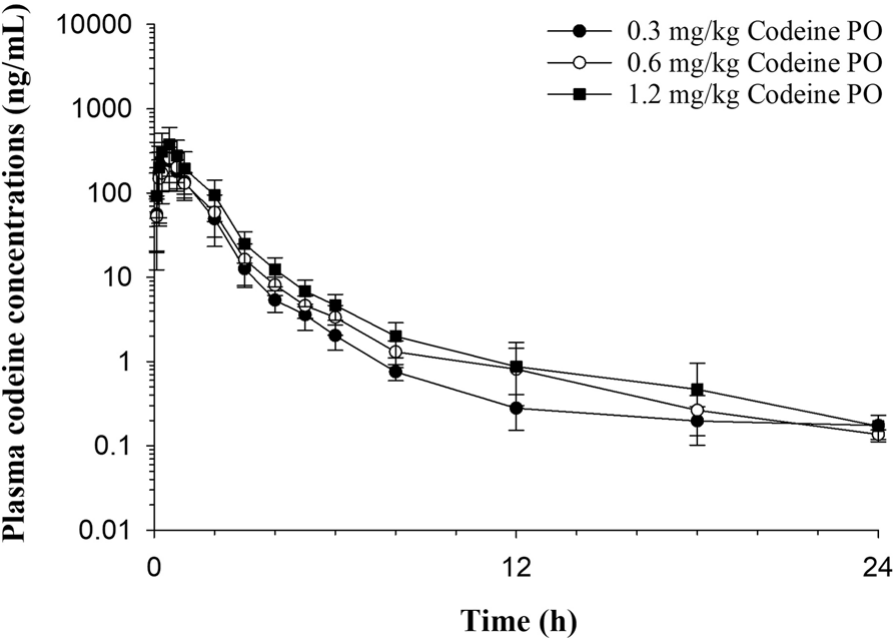
Mean ± SD plasma concentrations of codeine with respect to time after a single oral administration of codeine (0.3, 0.6 and 1.2 mg/kg) to seven horses

**Fig. 2:**
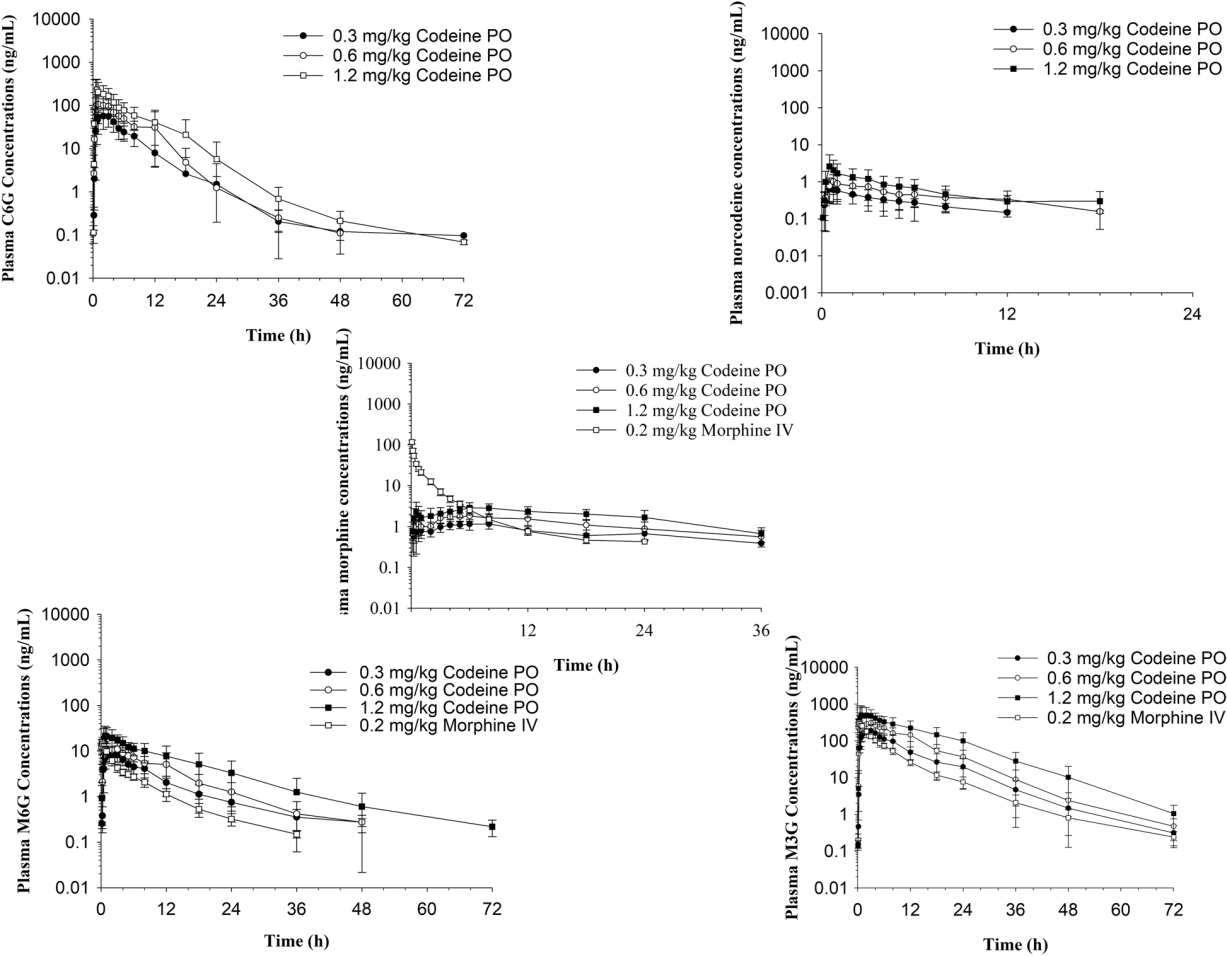
Mean ± SD plasma concentrations of codeine-6-glucuronide (C6G), norcodeine, morphine, morphine-6-glucuronide (M6G) and morphine-3-glucuronide (M3G) with respect to time after a single oral administration of codeine (0.3, 0.6 and 1.2 mg/kg) or intravenous administration of morphine (0.2 mg/kg) to seven horses

Codeine concentration time curves are depicted in Fig. 1. Following administration of all doses, codeine was below the limit of detection of the analytical assay by 24 hours post administration. Codeine was rapidly metabolized to several metabolites. At all doses, the most abundant metabolite was M3G, followed by C6G, M6G, morphine and norcodeine (Fig. 2). Except for the later time points (post 6 hours), morphine concentrations following intravenous administration were significantly higher than that observed in all codeine dose groups (Table 1A). Morphine-6-glucuronide concentrations were significantly higher in the morphine dose group compared to all codeine groups until 15 minutes post administration (Table 1B). Concentrations of M6G were significantly higher in the 1.2 mg/kg codeine dose group, compared to the morphine group starting at 1 hour post drug administration until 72-hours following administration (Table 1B) and from 3 to 24 hours and 3 to 5 hours following administration of 0.6 and 0.3 mg/kg codeine, respectively (Table 1B). Concentrations of M3G were significantly higher following morphine administration compared to all codeine dose groups until 15 minutes post drug administration (Table 1C). Morphine-3-glucuronide concentrations were significantly higher following codeine administration, compared to the morphine dose group from 3 until 36 hours and from 2 until 72 hours post drug administration for the 0.6 and 1.2 mg/kg dose groups, respectively (Table 1C).

**Table 1:** Mean ± SD plasma concentrations of (A) morphine, (B) morphine-6-glucuronide (M6G) and (C) morphine-3-glucuronide (M3G) following a single oral administration of codeine (0.3, 0.6, 1.2 mg/kg) or intravenous administration of morphine (0.2 mg/kg) to 7 horses

	Morphine Concentration (ng/mL)			
	Codeine			Morphine
A)				
Time (h)	0.3 mg/kg PO	0.6 mg/kg PO	1.2 mg/kg PO	0.2 mg/kg IV
0	ND	ND	ND	ND
0.08	ND^d^	ND^d^	ND^d^	117.4 ± 12.7^,b,c^
0.16	ND	0.68 ± 0.22^d^	0.76 ± 0.52^d^	72.0 ± 9.76^a,b,c^
0.25	0.56 ± 0^d^	1.02 ± 0.83^d^	1.56 ± 1.17^d^	53.9 ± 7.23^a,b,c^
0.5	0.72 ± 0.51^d^	0.97 ± 0.45^d^	2.28 ± 1.71^d^	34.2 ± 3.91^a,b,c^
0.75	0.81 ± 0.38^d^	1.13 ± 0.36^d^	1.87 ± 1.24^d^	25.5 ± 3.34^a,b,c^
1.0	0.77 ± 0.26^d^	0.99 ± 0.39^d^	1.66 ± 0.80^d^	21.2 ± 3.35^a,b,c^
2.0	0.75 ± 0.19^d^	1.02 ± 0.27^d^	1.81 ± 0.99^d^	12.5 ± 2.09^a,b,c^
3.0	0.97 ± 0.25^d^	1.61 ± 0.44^d^	2.08 ± 0.86^d^	7.02 ± 1.28^a,b,c^
4.0	1.08 ± 0.25^d^	1.73 ± 0.61^d^	2.34 ± 0.84^d^	4.79 ± 0.88^a,b,c^
5.0	1.10 ± 0.21^c,d^	1.75 ± 0.40^d^	2.57 ± 0.90^a^	3.62 ± 0.59^a,b^
6.0	1.15 ± 0.34^c,d^	1.82 ± 0.47	2.88 ± 0.96^a^	2.56 ± 0.49^a^
8.0	1.16 ± 0.29^c^	1.64 ± 0.37^d^	2.84 ± 0.78^a,b,d^	1.49 ± 0.27^c^
12.0	0.80 ± 0.20^b,c^	1.54 ± 0.47^a,c,d^	2.35 ± 0.71^a,b,d^	0.76 ± 0.11^b,c^
18.0	0.60 ± 0.22^b,c^	1.09 ± 0.41^a,c,d^	2.02 ± 0.61^a,b,d^	0.46 ± 0.08^b,c^
24.0	0.67 ± 0.20^b,c^	0.88 ± 0.43^a,d^	1.68 ± 0.80^a,d^	0.43 ± 0.0^b,c^
36.0	0.39 ± 0^c^	0.56 ± 0.25^c^	0.67 ± 0.26^a,b,d^	ND^c^
48.0	ND	ND	0.66 ± 0.21	ND
72.0	ND	ND	ND	ND
B)				
	**Morphine 6-glucuronide Concentration (ng/mL)**			
	**Codeine**			**Morphine**
Time (h)	0.3 mg/kg PO	0.6 mg/kg PO	1.2 mg/kg PO	0.2 mg/kg IV
0	ND	ND	ND	ND
0.08	ND^d^	ND^d^	ND^d^	12.6 ± 2.31^a,b,c^
0.16	0.25 ± 0.02^d^	0.97 ± 0.78^d^	0.92 ± 1.14^d^	15.9 ± 2.10^a,b,c^
0.25	0.38 ± 0.22^c,d^	2.10 ± 3.04d	3.90 ± 4.48^d^	14.3 ± 3.27^a,b,c^
0.5	4.05 ± 2.84^c,d^	7.94 ± 8.22	18.3 ± 12.7^a^	12.4 ± 2.56^a^
0.75	6.93 ± 2.94^c^	10.5 ± 5.26	21.6 ± 13.3^a^	10.8 ± 2.15
1.0	8.25 ± 2.45^c^	10.7 ± 3.69^c^	20.7 ± 12.0^a,c,d^	9.77 ± 2.02^c^
2.0	8.13 ± 2.21^c^	10.3 ± 2.44^c^	19.4 ± 9.90^a,b,d^	6.14 ± 1.43^c^
3.0	8.19 ± 2.49^c,d^	10.9 ± 3.84^c,d^	17.8 ± 4.87^a,b,d^	4.27 ± 0.88^a,b,c^
4.0	6.43 ± 1.71^c,d^	8.83 ± 3.00^c,d^	14.9 ± 5.56^a,b,d^	3.43 ± 0.57^a,b,c^
5.0	5.07 ± 1.28^b,c,d^	7.78 ± 2.77^a,c,d^	12.2 ± 2.98^a,b,d^	3.06 ± 0.41^a,b,c^
6.0	4.46 ± 1.33^b,c^	7.28 ± 2,79^a,c,d^	11.3 ± 3.43^a,b,d^	2.69 ± 0.43^b,c^
8.0	4.12 ± 1.91^c^	5.38 ± 2.21^c,d^	10.0 ± 4.54^a,b,d^	2.08 ± 0.47^b,c^
12.0	2.04 ± 0.75^b,c^	5.08 ± 4.06^a,c,d^	7.71 ± 5.16^a,d^	1.13 ± 0.34^b,c^
18.0	1.12 ± 0.69^c^	1.96 ± 1.08^c,d^	5.10 ± 3.88^a,b,d^	0.53 ± 0.18^b,c^
24.0	0.75 ± 0.47^c^	1.25 ± 0.77^c,d^	3.32 ± 2.73^a,b,d^	0.32 ± 0.10^b,c^
36.0	0.35 ± 0.17_c_	0.42 ± 0.36^c^	1.25 ± 1.26^a,b,d^	0.15 ± 0.03^c^
48.0	0.27 ± 0.05	0.28 ± 0.11	0.60 ± 0.58^d^	ND^c^
72.0	ND	ND	0.22 ± 0.09^d^	ND^c^
C)				
	**Morphine 3-glucuronide Concentration (ng/mL)**			
	**Codeine**			**Morphine**
Time (h)	0.3 mg/kg PO	0.6 mg/kg PO	1.2 mg/kg PO	0.2 mg/kg IV
0	ND	ND	ND	ND
0.08	0.14 ± 0.02^d^	0.20 ± 0.09^d^	0.16 ± 0.04d	242.8 ± 74.3^a,b,c^
0.16	0.46 ± 0.24^d^	5.83 ± 9.74^d^	5.09 ± 8.13^d^	324.3 ± 73.6^a,b,c^
0.25	3.46 ± 2.24^d^	44.9 ± 81.6^d^	63.4 ± 85.2^d^	319.8 ± 63.4^a,b,c^
0.5	65.0 ± 70.4^c,d^	196.9 ± 263.0	422.3 ± 374.9^a^	291.3 ± 51.6^a^
0.75	119.7 ± 61.6^c^	265.5 ± 194.6	505.5 ± 413.0^a^	266.4 ± 40.1
1.0	144.63 ± 47.2^c^	265.3 ± 141.4	493.6 ± 255.4^a^	255.1 ± 33.4
2.0	170.7 ± 47.9^c^	284.9 ± 136.3^c^	499.1 ± 310.1^a,b,d^	181.0 ± 21.7^c^
3.0	188.1 ± 68.0^b,c^	303.3 ± 108.2^a,c,d^	490.1 ± 207.4^a,b,d^	131.9 ± 18.3^b,c^
4.0	161.4 ± 50.7^b,c^	266.5 ± 95.5^a,c,d^	418.3 ± 143.1^a,b,d^	103.0 ± 16.3^b,c^
5.0	126.7 ± 39.0^c^	236.4 ± 78.2^a,c,d^	357.6 ± 118.8^a,b,d^	85.0 ± 17.7^b,c^
6.0	110.6 ± 42.2^b,c^	224.0 ± 85.7^a,c,d^	329.5 ± 121.9^a,c,d^	73.6 ± 12.7^b,c^
8.0	96.5 ± 42.4^b,c^	161.8 ± 64.4^a,c,d^	287.6 ± 140.1^a,b,d^	52.9 ± 10.5^b,c^
12.0	48.6 ± 20.0^b,c^	144.3 ± 90.2^a,d^	223.2 ± 126.3^a,d^	26.0 ± 4.98^b,c^
18.0	26.43 ± 18.0^b,c^	53.9 ± 25.6^a,c,d^	146.5 ± 83.0^a,b,d^	11.7 ± 2.58^b,c^
24.0	19.7 ± 14.6^c^	36.8 ± 19.0^c,d^	100.0 ± 67.2^a,b,d^	7.60 ± 2.81^b,c^
36.0	4.67 ± 4.23^c^	8.87 ± 7.17^c,d^	28.2 ± 20.0^a,b,d^	2.08 ± 1.27^b,c^
48.0	1.47 ± 1.20^c^	2.39 ± 1.52^c^	10.2 ± 10.1^a,b,d^	0.81 ± 0.54^c^
72.0	0.31 ± 0.19^c^	0.47 ± 0.28^c^	1.05 ± 0.71^a,b,d^	0.24 ± 0.10^c^

*ND* not detected

a, significantly different (*p* < 0.05) from 0.3 mg/kg; b significantly different (*p* < 0.05) from 0.6 mg/kg; c, significantly different (*p* < 0.05) from 1.2 mg/kg; d, significantly different (*p* < 0.05) from 0.2 mg/kg morphine

Pharmacokinetic parameters are listed in Tables 2 and 3 for codeine and morphine, respectively and Table 4 for M6G, M3G, C6G and norcodeine. No significant difference in pharmacokinetic parameters for codeine were observed between dose groups (Table 2). C_max_ and AUC for codeine did not increase in a proportionate manner with higher codeine doses. A significant difference was observed in the morphine C_max_ values between all groups (Table 3). Maximum concentrations of M6G were significantly higher in the 1.2 mg/kg codeine dose group compared to all other groups (codeine and morphine; Table 4). The M6G AUC was significantly higher in the 0.6 and 1.2 mg/kg codeine dose groups compared to the morphine group (Table 4). A significant difference in M3G C_max_ was noted between all codeine dose groups but none were significantly different from the morphine dose group (Table 4). The M3G AUC was significantly different between all groups (codeine and morphine). The C6G C_max_ and AUC values increased in a dose-proportionate manner with increasing codeine doses (Table 4). No other significant differences in pharmacokinetic parameters between codeine dose groups were observed. For norcodeine, the AUC value increased in a dose-proportionate manner (Table 4).

**Table 2:** Pharmacokinetic parameters (geometric mean or median (Tmax) and range) for codeine following a single oral administration of codeine (0.3, 0.6 and 1.2 mg/kg) to adult horses. All values reported were generated using non-compartmental analysis

Parameters	Dose Groups		
	0.3 mg/kg (*n* = 7)	0.6 mg/kg (*n* = 7)	1.2 mg/kg (*n* = 7)
C_max_ ng/mL	266.0 (91.2–415.4)	242.8 (132.1–551.0)	347.8 (125.4–693.0)
T_max_ (h)	0.5 (0.16–1.0)	0.5 (0.5–0.75)	0.5 (0.25–1.0)
Lambda_z_(1/h)	0.345 (0.178–0.695)	0.328 (0.151–0.654)	0.252 (0.130–0.418)
HL Lambda_z_ (h)^*^	1.84 (1.00–3.90)	1.94 (1.06–4.59)	2.60 (1.66–5.32)
AUC_0-inf_ (h*ng/mL)	295.4 (157.1–456.3)	308.7 (185.7–502.2)	457.6 (189.0–934.6)

*, harmonic mean

*C*_*max*_ maximum concentration, *T*_*max*_ time to C_max_, *Lambda*_*z*_ terminal slope, *HL Lambda*_*z*_ terminal half-life, *AUC*_*0-inf*_ area under the plasma-concentration curve from time 0 to infinity

a, significantly different (*p* < 0.05) from 0.3 mg/kg; b significantly different (*p* < 0.05) from 0.6 mg/kg; c, significantly different (*p* < 0.05) from 1.2 mg/kg

**Table 3:** Pharmacokinetic parameters (geometric mean or median (T_max_) and range) for morphine following a single oral administration of codeine (0.3, 0.6 and 1.2 mg/kg) or a single IV administration (0.2 mg/kg) of morphine to adult horses. All values reported were generated using non-compartmental analysis

Parameters	Dose Groups			
	Codeine 0.3 mg/kg PO (*n* = 7)	Codeine 0.6 mg/kg PO (*n* = 7)	Codeine 1.2 mg/kg PO (*n* = 7)	Morphine 0.2 mg/kg IV (*n* = 7)
C(0) ng/mL	–	–	–	191.1 (167.3–235.0)
C_max_ ng/mL	1.26 (0.855–1.74)^bc^	2.11 (1.50–3.03)^ac^	3.37 (2.16–4.50)^ab^	–
T_max_ (h)	6.0 (0.5–8.0)	6.0 (0.25–12.0)	7.0 (0.5–24.0)	–
Lambda_z_(1/h)	0.048 (0.015–0.089)	0.052 (0.027–0.072)	0.067 (0.046–0.089)	0.138 (0.036–0.215)
HL Lambda_z_ (h)^*^	12.3 (7.76–46.2)	12.6 (9.56–26.0)	10.0 (7.82–15.2)	5.17 (3.22–19.4)
Vdss (L/kg)	–	–	–	6.72 (4.36–11.9)
CL (mL/h/kg)	**–**	–	–	1967 (1584–2448)
AUC_0-inf_ (h*ng/mL)	29.7 (15.4–91.4)^c^	48.4 (36.1–82.3)^c^	74.5 (57.2–111.4)^abd^	101.7 (81.7–126.2)^c^

*, harmonic mean; −--, NA

*C(0)* concentration extrapolated to the origin, *C*_*max*_ maximum concentration, *T*_*max*_ time to C_max_, *Lambda*_*z*_ terminal slope, *HL Lambda*_*z*_ terminal half-life, *Vdss* Volume of distribution at steady-state, *CL* clearance, *AUC*_*0-inf*_ area under the plasma-concentration curve from time 0 to infinity

a, significantly different (*p* < 0.05) from 0.3 mg/kg; b significantly different (*p* < 0.05) from 0.6 mg/kg; c, significantly different (*p* < 0.05) from 1.2 mg/kg; d, significantly different (*p* < 0.05) from 0.2 mg/kg morphine

**Table 4:** Pharmacokinetic parameters (geometric mean or median (T_max_) and range) for morphine 3-glucuronide following a single administration of codeine (0.3, 0.6 and 1.2 mg/kg oral) or morphine (0.2 mg/kg IV) to adult horses. All values reported were generated using non-compartmental analysis

	C_max_ (ng/mL)	T_max_ (h)	Lambda_z_ (1/h)	HL Lambda_z_ (h)^*^	AUC_0-inf_ (h*ng/mL)
0.3 mg/kg Codeine PO (*n* = 7)					
M6G	9.55 (6.82–12.8)^c^	3.0 (0.75–3.0)	0.102 (0.056–0.158)	6.37 (4.38–12.5)	79.0 (49.3–113.9)
M3G	199.9 (130.2–320.6)^bc^	3.9 (0.75–4.0)	0.104 (0.087–0.154)	6.58 (4.51–7.96)	1782 (1251–2839)^bcd^
C6G	68.6 (32.8–101.9) ^bc^	2.0 (0.5–3.0)	0.181 (0.104–0.332)	3.54 (2.09–6.65)	383.6 (263.2–665.6)^bc^
Norcodeine	0.62 (0.37–1.32)^c^	0.75 (0.75–1.0)	0.258 (0.152–0.487)^bc^	2.44 (1.42–4.55)^bc^	2.53 (1.04–6.69)^bc^
0.6 mg/kg Codeine PO (*n* = 7)					
M6G	14.0 (17.0–23.3)^c^	2.0 (0.5–12.0)	0.092 (0.046–0.122)	7.24 (5.66–14.9)	123.6 (71.3–211.0)^cd^
M3G	370.8 (235.9–760.1)^ac^	2.5 (0.5–12.0)	0.094 (0.063–0.120)	7.23 (5.79–11.0)	3540 (2780–4608)^acd^
C6G	129.6 (56.4–254.6) ^ac^	2.5 (0.5–12.0)	0.174 (0.120–0.298)	3.76 (2.32–5.77)	729.6 (420.7–1032)^ac^
Norcodeine	0.88 (0.49–1.68)	0.75 (0.5–3.0)^c^	0.149 (0.051–0.357)	3.93 (1.94–13.7)^a^	5.55 (2.58–13.7)^ac^
1.2 mg/kg Codeine PO (*n* = 7)					
M6G	24.4 (14.0–44.3)^abd^	1.5 (0.5–12.0)	0.091 (0.055–0.123	7.38 (5.64–12.5)	238.3 (165.0–436.0)^abd^
M3G	579.4 (277.5–1265.3)	2.5 (0.5–12.0)	0.093 (0.079–0.113)	7.42 (6.16–8.73)	6710 (4993–9631)^abd^
C6G	219.4 (90.9–481.0) ^ab^	1.4 (0.5–12.0)	0.151 (0.096–0.221)	4.43 (3.14–7.22)	1346 (663.3–2081)^ab^
Norcodeine	1.48 (0.59–7.34)^a^	0.5 (0.25–2.0)^b^	0.123 (0.026–0.215)^a^	4.26 (1.79–27.0)^a^	10.8 (3.62–32.1)^ab^
0.2 mg/kg Morphine IV					
M6G	14.5 (8.17–17.9)	0.16 (0.16–0.25)	0.118 (0.081–0.152)	5.78 (4.56–8.58)	54.5 (38.7–74.1)^bc^
M3G	320.3 (214.2–400.6)	0.16 (0.16–0.75)	0.079 (0.046–0.114)	8.41 (6.07–15.0)	1438 (1130–1706)^abc^

*, harmonic mean; *C*_*max*_ maximum concentration, *T*_*max*_ time to C_max_, *Lambda*_*z*_ terminal slope, *HL Lambda*_*z*_ terminal half-life, *AUC*_*0-inf*_ area under the plasma-concentration curve from time 0 to infinity

a, significantly different (*p* < 0.05) from 0.3 mg/kg; b significantly different (*p* < 0.05) from 0.6 mg/kg; c, significantly different (*p* < 0.05) from 1.2 mg/kg; d, significantly different (*p* < 0.05) from 0.2 mg/kg morphine

### Behavioral and physiologic effects

Following administration of 0.3 mg/kg and 0.6 mg/kg, one horse (the same horse) exhibited signs of colic, including agitation, pawing, and turning his head to look at his stomach. The horse was randomized to the 0.6 mg/kg dose group first and subsequently to the 0.3 mg/kg group. Following administration of the 0.6 mg/kg dose, signs began at 15 minutes and had resolved by 1 hour post administration. Following the 0.3 mg/kg dose, signs of colic persisted for 24 hours post administration. No adverse effects were noted in the other six horses studied at any dose of codeine. Following morphine administration, four horses displayed signs of agitation, including pawing at the ground, and circling within the first 2 minutes of drug administration. Three of these 4 horses exhibited trembling, starting in the flanks and progressing to full body tremors within 15 to 45 minutes. Agitation and trembling had resolved in all horses by 4 hours post morphine administration.

**Fig. 3:**
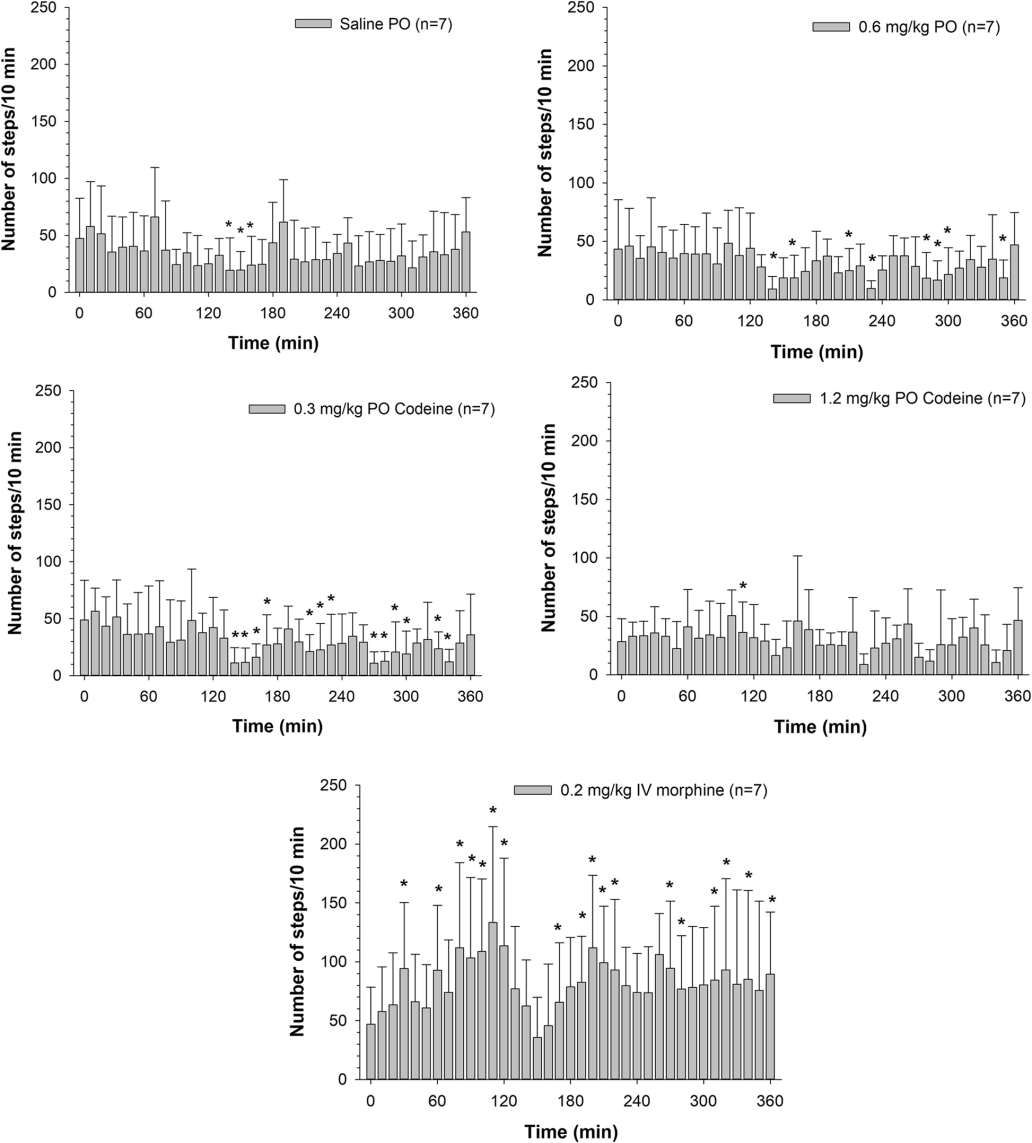
Mean ± SD number of Steps taken (over a 10-minute period of time) with respect to time following a single oral administration of codeine (0.3, 0.6 and 1.2 mg/kg) or intravenous administration of morphine (0.2 mg/kg) to seven horses. *Significant differences relative to time 0 (*p* < 0.05)

Significant changes in the number of steps, relative to baseline, were noted in the saline, and 0.3 and 0.6 mg/kg codeine dose groups at various time points starting around 2 hours post administration (Fig. 3). Significant increases in the number of steps were noted following morphine administration starting at 30 minutes post drug administration (Fig. 3). Heart rates prior to and post administration of saline, codeine and morphine are listed in Table 5.

**Table 5:** Heart rate (mean ± SD bpm), following a single oral administration of saline, single oral administration of codeine (0.3, 0.6 and 1.2 mg/kg) or a single intravenous administration of morphine (0.2 mg/kg) to 7 adult horses

Time (h)	Saline	Codeine 0.3 mg/kg PO (*n* = 7)	Codeine 0.6 mg/kg PO (*n* = 7)	Codeine 1.2 mg/kg PO (*n* = 7)	Morphine 0.2 mg/kg IV (*n* = 7)
0	36 ± 7	40 ± 9	35 ± 5	37 ± 5	40 ± 8
0.03	40 ± 5	40 ± 9	38 ± 7	40 ± 5^*^	42 ± 7
0.08	37 ± 5	36 ± 7^*^	37 ± 4	35 ± 5	46 ± 8^*^
0.13	38 ± 6	38 ± 10	40 ± 11^*^	40 ± 9	50 ± 11^*^
0.17	36 ± 7	37 ± 7	36 ± 8	35 ± 5	44 ± 5
0.20	46 ± 32	39 ± 9	35 ± 7	37 ± 5	47 ± 10^*^
0.25	41 ± 14	37 ± 8	37 ± 7	35 ± 4	51 ± 14^*^
0.33	38 ± 8	38 ± 11	42 ± 14^*^	37 ± 7	48 ± 9^*^
0.5	40 ± 12	36 ± 8^*^	36 ± 6	35 ± 5	41 ± 5
0.75	38 ± 7	35 ± 8^*^	34 ± 4	35 ± 4	39 ± 5
1	38 ± 7	35 ± 6^*^	36 ± 6	34 ± 5	40 ± 4^*^
1.25	35 ± 7	32 ± 8^*^	34 ± 6	34 ± 6	48 ± 8
1.5	37 ± 7	36 ± 7	38 ± 8	36 ± 6	44 ± 4
2	39 ± 7	34 ± 6^*^	33 ± 4	32 ± 5	41 ± 4^*^
2.5	40 ± 5	40 ± 6	40 ± 7^*^	43 ± 12^*^	48 ± 7
3	39 ± 6	35 ± 5	35 ± 4	37 ± 9	44 ± 5
4	38 ± 6	36 ± 8	37 ± 5	33 ± 4	42 ± 6
5	35 ± 7	37 ± 8	35 ± 3	38 ± 11	41 ± 4
6	41 ± 6	37 ± 6	37 ± 7	37 ± 6	41 ± 5

*, indicates a significant difference (*p* < 0.05) relative to baseline

Gastrointestinal scores were not significantly decreased relative to baseline at any time post administration in any dose group (Table 6). Overall, there was not a noticeable difference in the number of fecal piles, size of the fecal balls within a pile or consistency between dose groups.

**Table 6:** Gastrointestinal scores (mean ± SD), following a single oral administration of saline, single oral administration of codeine (0.3, 0.6 and 1.2 mg/kg) or a single intravenous administration of morphine (0.2 mg/kg) to 7 adult horses

Time (h)	Saline	Codeine 0.3 mg/kg PO (*n* = 7)	Codeine 0.6 mg/kg PO (*n* = 7)	Codeine 1.2 mg/kg PO (*n* = 7)	Morphine 0.2 mg/kg IV (n = 7)
Baseline	2.7 ± 0.8	2.7 ± 8	1.7 ± 0.5	2.4 ± 1.3	2.4 ± 1.5
0.5	3.4 ± 1.5	3.0 ± 0.8	2.3 ± 1.0	2.6 ± 0.8	1.4 ± 1.4
0.75	3.4 ± 1.3	3.0 ± 0.6	1.9 ± 0.7	2.0 ± 0.6	1.9 ± 1.7
1	3.1 ± 1.3	2.7 ± 1.8	2.6 ± 0.8	1.7 ± 0.8	1.6 ± 0.5
2	3.9 ± 0.7^*^	2.9 ± 0.7	2.7 ± 1.0	2.4 ± 0.8	2.1 ± 1.3
4	3.9 ± 0.7^*^	3.6 ± 1.0	4.0 ± 0.8^*^	3.4 ± 0.8	3.9 ± 1.1^*^
6	3.7 ± 1.0^*^	3.3 ± 0.8	3.4 ± 0.8^*^	3.4 ± 1.1	3.4 ± 1.4
24	–	4.0 ± 0.8^*^	3.3 ± 1.7^*^	3.0 ± 1.5	3.3 ± 0.5

---, not assessed; *, indicates a significant difference (*p* < 0.05) relative to baseline

### Thermal nociception

Table 7 lists skin and ambient temperature, thermal threshold and the thermal exclusion (%TE) at each time point post saline or drug administration. A significant increase in the thermal threshold (T_T_) was noted at 6 hours post administration of 0.6 mg/kg codeine and a significant increase in the %TE at 2 hours post administration of 0.3 mg/kg codeine (Table 7).

**Table 7:** Skin temperature, thermal threshold (TT) and thermal excursion (%TE) and ambient temperature following a single oral administration of saline, 0.3, 0.6 and 1.2 mg/kg codeine and intravenous 0.2 mg/kg morphine to 7 horses. Values are expressed as mean ± SD

Dose Group	Time (h)									
	0	0.25	0.5	0.75	1	1.5	2	3	4	6
Skin Temp (°C)										
Saline	27.4 ± 2.2	27.8 ± 2.2	27.4 ± 2.4	27.8 ± 2.6	28.3 ± 2.4	28.8 ± 1.7	29.5 ± 1.7*	30.9 ± 0.5*	31.0 ± 0.6*	31.3 ± 1.1*
0.3 mg/kg	27.6 ± 3.5	27.1 ± 3.1	27.7 ± 3.3	28.3 ± 3.9	28.8 ± 3.9	29.3 ± 3.0*	29.1 ± 3.3	30.7 ± 1.8*	31.6 ± 0.7*	31.9 ± 1.0*
0.6 mg/kg	28.2 ± 1.5	28.7 ± 1.3	28.3 ± 2.2	28.4 ± 2.8	28.4 ± 2.7	28.8 ± 2.4	28.8 ± 1.9	31.3 ± 1.1*	31.2 ± 0.9*	31.9 ± 0.6*
1.2 mg/kg	26.8 ± 3.7	26.8 ± 3.7	27.2 ± 4.1	27.9 ± 4.3	28.8 ± 3.2*	28.8 ± 3.1*	29.9 ± 2.8*	31.3 ± 1.5*	31.5 ± 1.2*	32.1 ± 0.6*
Morphine	26.8 ± 3.6	26.0 ± 3.3	25.9 ± 2.9	27.2 ± 3.0	28.0 ± 3.3	28.4 ± 3.8	28.8 ± 3.8*	30.4 ± 4.1*	30.3 ± 3.5*	31.1 ± 0.8*
TT (°C)										
Saline	48.5 ± 4.4	44.7 ± 3.0*	46.0 ± 3.5	45.4 ± 2.8	47.6 ± 4.9	46.4 ± 3.3	47.0 ± 3.7	49.3 ± 4.2	48.4 ± 4.0	50.3 ± 5.1
0.3 mg/kg	48.8 ± 4.6	46.9 ± 6.9	50.0 ± 5.5	46.7 ± 7.5	48.5 ± 9.2	47.8 ± 5.1	51.9 ± 4.6	50.2 ± 6.1	49.6 ± 5.2	50.6 ± 4.3
0.6 mg/kg	48.0 ± 4.8	46.1 ± 5.3	45.9 ± 6.2	48.9 ± 4.7	47.3 ± 4.4	48.9 ± 5.3	48.2 ± 4.7	50.4 ± 5.1	48.5 ± 5.9	52.1 ± 3.8*
1.2 mg/kg	48.8 ± 4.3	47.5 ± 4.7	49.7 ± 4.1	48.8 ± 4.5	47.5 ± 5.3	49.5 ± 3.1	50.4 ± 4.1	48.5 ± 2.6	50.5 ± 4.1	49.4 ± 4.5
Morphine	48.3 ± 4.5	49.6 ± 5.4	50.8 ± 3.6	50.6 ± 3.8	49.6 ± 4.2	50.0 ± 4.1	50.2 ± 4.9	50.8 ± 3.0	50.6 ± 4.6	49.2 ± 2.5
% TE										
Saline	75.5 ± 17.2	60.8 ± 11.2	66.4 ± 11.3	66.4 ± 10.1	70.1 ± 18.5	65.3 ± 14.5	67.4 ± 13.3	74.9 ± 16.7	71.1 ± 16.6	79.2 ± 17.3
0.3 mg/kg	77.3 ± 14.4	70.5 ± 21.7	81.2 ± 16.9	68.4 ± 23.8	76.4 ± 29.2	71.1 ± 19.3	87.2 ± 17.1*	78.7 ± 25.0	75.3 ± 21.6	79.5 ± 17.3
0.6 mg/kg	73.0 ± 17.6	65.4 ± 19.0	65.6 ± 20.1	74.8 ± 18.4	69.7 ± 17.4	75.4 ± 20.8	72.8 ± 17.6	79.2 ± 21.3	71.1 ± 24.7	86.3 ± 15.3
1.2 mg/kg	77.0 ± 15.9	73.1 ± 17.9	79.5 ± 14.2	75.2 ± 16.8	69.5 ± 20.6	77.3 ± 11.8	79.6 ± 17.4	70.6 ± 11.3	79.7 ± 17.1	73.7 ± 19.3
Morphine	75.2 ± 15.0	80.7 ± 19.0	84.8 ± 12.3	83.4 ± 13.4	79.7 ± 14.4	80.3 ± 15.7	81.7 ± 17.3	82.6 ± 13.5	81.9 ± 17.0	75.9 ± 13.6
Ambient Temp (°C)										
Saline	20.0 ± 1.2	20.1 ± 1.3	20.2 ± 1.4	20.3 ± 1.4	20.3 ± 1.4	20.8 ± 1.4*	21.1 ± 1.4*	21.9 ± 1.1*	22.8 ± 0.9*	24.3 ± 1.3*
0.3 mg/kg	19.1 ± 1.3	19.3 ± 1.3	19.4 ± 1.4	19.5 ± 1.4*	19.7 ± 1.5*	20.0 ± 1.7*	20.1 ± 1.8*	21.1 ± 2.0*	22.3 ± 1.8*	24.6 ± 1.8*
0.6 mg/kg	19.4 ± 1.6	19.6 ± 1.7	19.8 ± 1.7	19.9 ± 1.6	20.0 ± 1.6*	20.4 ± 1.6*	20.5 ± 1.8*	21.6 ± 1.5*	22.6 ± 1.4*	24.8 ± 1.9*
1.2 mg/kg	19.5 ± 1.9	19.7 ± 1.8	19.7 ± 1.8	19.9 ± 1.8	20.1 ± 1.9	20.5 ± 1.9*	20.8 ± 2.1*	21.7 ± 2.1*	22.6 ± 1.9*	24.5 ± 1.8*
Morphine	19.1 ± 1.7	19.3 ± 1.5	19.4 ± 1.5	19.7 ± 1.5	19.9 ± 1.6*	20.4 ± 1.7*	20.9 ± 1.8*	21.7 ± 1.8*	22.6 ± 1.6*	23.5 ± 1.7*

## Discussion

In the current study, the metabolic profile, pharmacokinetics and pharmacodynamic effects, including effects on thermal nociception, following administration of three doses of codeine to horses is described. As described in a previous study [14], in the current study, codeine was rapidly metabolized to C6G and morphine with subsequent glucuronidation of the latter to M3G and M6G. At all doses, M3G concentrations far exceeded those of M6G, in agreement with previous studies describing the metabolism of both codeine and morphine in horses [14, 16, 19, 20].

To the best of the authors’ knowledge, there is only a single previous report describing the pharmacokinetics of codeine in horses [14]. Gretler and colleagues [14] chose a dose of 0.6 mg/kg and pharmacokinetic parameters calculated following administration of 0.6 mg/kg in the current study agreed with those reported previously. With the inclusion of additional doses in the current study, however, a less than proportional increase in AUC_0-inf_ and C_max_ of codeine was observed with increasing dose. This less than proportional increase in AUC has been reported for several drugs following oral administration and is most commonly attributed to either dose-dependent absorption or an increase in elimination [21]. One of the more common causes of dose-dependent absorption is limited solubility [21] and while this is a possible explanation for the apparent disproportionate absorption of codeine at higher doses, in general codeine is very soluble in the acidic contents of the GI tract and so is unlikely to be a factor in the current study. Another reason for dose dependent absorption is a decrease in movement across intestinal epithelial cells because of saturation of transport proteins involved in drug absorption [21]. This is a less likely explanation as these transport proteins tend to be high capacity and in the case of codeine, to the best of the authors’ knowledge, there is no evidence of transport proteins playing a role in absorption of this compound.

A more likely explanation for the disproportionate increase in AUC and C_max_ with increasing codeine dose is an increase in metabolism. The increase in metabolite concentrations with increasing dose along with a dose-proportionate increase in C_max_ and AUC values for the metabolites suggest increased biotransformation of codeine. Codeine, as well as morphine, are highly susceptible to the first pass effect [22–24], and it is plausible that pre-systemic (GIT and/or hepatic) metabolism may explain the less than proportionate increase in the AUC and C_max_ of codeine. Intravenous administration of codeine has not been reported for horses, but the relative concentrations of parent drug and metabolite following administration of multiple doses by this route of administration may offer insight into whether the first pass effect results in an increase in biotransformation of codeine following oral administration, explaining the disproportionate increase in codeine AUC and C_max_. Additionally, since morphine is a major metabolite of codeine, studies describing the pharmacokinetics of morphine following oral administration to horses may help to elucidate the role of the first pass effect in the apparent lack of dose dependent change in AUC.

A large degree of variation in pharmacokinetic parameters (i.e. terminal half-life and AUC) for codeine and its metabolites was noted between horses. While it is not possible to definitely determine the reason for this high degree of variability between horses, this has been reported previously for codeine and morphine in horses [14, 19]. One possible explanation for the highly variable terminal half-life and AUC is differing metabolic activity between horses. In horses, CYP2D82, an orthologue to human CYP2D6 is responsible for the biotransformation of codeine to morphine [25] and similar to what has been described for CYP2D6 in humans, the existence of polymorphisms in the gene that codes for this enzyme have been suggested [26]. In humans, these polymorphisms can lead to altered (poor or extensive) metabolism of compounds, such as codeine, that are substrates for this enzyme [3, 27]. Although to date there are no published reports in horses, similar to CYP2D6, polymorphisms have been identified in human glucuronosyl transferase (UGT) enzymes, leading to altered glucuronidation and generation of compounds such as M3G, M6G and C6G [28]. If such polymorphisms exist in horses, this may explain the variability observed in the terminal half-life and AUC for the glucuronidated metabolites in the current study.

With the exception of one horse, codeine administration was well tolerated at all doses studied. One horse exhibited colic like signs following administration of 0.3 and 0.6 mg/kg codeine. The reason for this reaction in a single horse is not immediately evident but it should be noted that interestingly this response was not observed in that horse at the highest dose (1.2 mg/kg). In contrast to the responses seen following codeine administration, several adverse behavioral effects were noted following morphine administration including agitation, tremors, and circling. Concurrent with these behavioral effects, a significant increase in heart rate, compared to pre-morphine administration was noted. These responses are similar to what has been reported previously at this dose [16, 19, 20]. Studies in other species, have suggested that the M3G metabolite may lead to neuroexcitation, and other adverse effects associated with morphine administration [29]. In the current study, significantly higher concentrations of M3G were observed following codeine administration compared to morphine administration. While administration of M3G would be necessary to definitively determine the behavioral and physiologic effects of this compound in the horse, the lack of notable adverse effects in the codeine group along with significantly higher concentrations of M3G as that seen following morphine administration, suggest that M3G may not be responsible for the adverse effects noted following morphine administration at the dose utilized in the current study. However, it is also important to remember that several metabolites are produced following codeine administration and it is also plausible that the apparent lack of excitation in horses may be a result of “masking” of M3G induced neuroexcitation by one of these metabolites (i.e. C6G). Similar to what was described previously, administration of M3G to horses would be necessary to determine if this occurs.

Codeine’s anti-nociceptive effects have been predominately attributed to the generation of morphine [4]. In turn, the analgesic effects of morphine, at least in part, have been attributed to M6G in some species [5–8]. It has also been suggested in some species that C6G may also contribute to the anti-nociceptive effects of codeine [9–11]. Codeine doses in the current study were selected based on what was determined most likely to achieve morphine and M6G concentrations equivalent to that achieved following intravenous administration of 0.2 mg/kg, a reported therapeutic dose [30, 31], using data from a previously published codeine pharmacokinetic study in horses [14]. It was hypothesized that codeine administration at these doses would then result in comparable anti-nociceptive effects to morphine. With respect to morphine, concentrations were low following codeine administration and were significantly different than that achieved following intravenous administration of morphine. This is likely a result of metabolism of codeine to C6G and/or pre-systemic glucuronidation of the morphine metabolite. Following administration of 0.3 mg/kg codeine, concentrations of M6G were higher or not significantly different from concentrations achieved following morphine administration, starting at 45 minutes post administration. Albeit at slightly later time points, maximum M6G concentrations following administration of 0.6 mg/kg codeine were comparable to that observed following intravenous morphine administration and higher following administration of 1.2 mg/kg of codeine. Concentrations in the codeine groups exceeded those observed in the morphine group starting at 1 hour post drug administration in the 0.6 and 1.2 mg/kg dose groups and at 2 hours in the 0.3 mg/kg group. Regardless of dose, there was not a significant thermal anti-nociceptive effect at any of the codeine doses studied.

In other species, most of the analgesic effects of morphine at the early times post administration have been attributed to morphine, as morphine more readily crosses the blood brain barrier reaching the target site more quickly compared to M6G [32, 33]. Therefore, the lack of an anti-nociceptive effect at the early times post codeine administration in the current study may be due to the low morphine concentrations because of rapid metabolism of morphine to M6G as part of the first pass effect. Furthermore, reports describing the analgesic effects of morphine suggest that M6G plays a greater role in analgesia with repeated administration of morphine as M6G accumulates at the target site in this situation [34, 35].

It is also important to note that although a previously published study reported a short-lived (up to 4 hours post administration) but significant thermal anti-nociceptive effect following administration of 0.2 mg/kg morphine [29], an anti-nociceptive effect was not observed following administration of the same dose of morphine in the current study. The reason for the discrepancy in morphine effects between the previous and current study is not immediately obvious.

The lack of effect on nociception following administration of 0.2 mg/kg morphine in the current study also contradicts with reports of analgesic effects in clinical cases whereby a dose of 0.2 mg/kg is considered within the effective range for eliciting analgesia [31]. This discrepancy highlights two important considerations when interpreting results from studies using experimental models of nociception and pain in normal horses and attempting to extrapolate to clinical cases. The first consideration is that normal horses may react differently than animals that are experiencing pain. It is possible that the antinociceptive effects of morphine were masked by the excitation elicited by drug administration in the clinically normal horses studied here. The same excitatory response may be less likely in a clinically ill horse. Secondly, although experimental models, such as the thermal nociceptive model used in the current study have been well described and allow for a controlled assessment, this may not be reflective of all clinical scenarios whereby a horse may be experiencing other types of pain (i.e. of inflammatory origin). Therefore, study of other types of anti-nociception and pain in horses is warranted.

## Conclusions

Although a thermal anti-nociceptive response was not observed following administration of codeine and morphine at the doses studied here, this study adds to and provides valuable information about the metabolism and pharmacokinetics of codeine and morphine in horses. Furthermore, it provides information that can be utilized in the design of future studies to understand the anti-nociceptive and analgesic effects of opioids in horses with the goal of promoting judicious and safe use of this important class of drugs.

## Methods

### Horses

Seven healthy university-owned, Thoroughbred horses (4 mares, 3 geldings, age: 6–10 years; weight: 452–670 kg) were studied. The number of horses selected was based on the thermal threshold. Power analysis, assuming a mean baseline thermal excursion of 71, a mean thermal excursion of 95 in each treatment group immediately after treatment and a mean thermal excursion of 85 in each treatment group 1-hour post treatment was conducted, as described in a previous study [36]. For paired t-tests, a standard deviation of 10 for the difference between baseline and treatment was used. Based on these assumptions, a sample size of 4 horses was deemed sufficient to detect a difference between baseline and immediately after treatment, and a sample size of 7 horses sufficient to detect a difference between baseline and 1-hour post-treatment.

Horses did not receive any medications for a minimum of four weeks prior to commencement of the study. Before beginning the study, horses were determined healthy by physical examination, complete blood count (CBC) and a serum biochemistry panel. Blood analyses were performed by the Clinical Pathology Laboratory of the William R. Pritchard Veterinary Medical Teaching Hospital of the University of California, Davis, using their standard protocols. Two days prior to the start of the study horses were moved into 12 × 12 stalls in a temperature-controlled barn. Horses remained in the stalls for a minimum of 48 hours following drug administration. The breezeway doors remained closed throughout the duration of the study and personnel access was limited to decrease external factors that might influence horse behavior. The study was conducted in accordance with the Institutional Animal Care and Use Committee of the University of California at Davis (protocol #22110).

### Instrumentation and drug administration

This study was conducted in a randomized balanced five-way balanced crossover design. Horses received a single oral administration of 0.9% NaCl (5 mL; negative control), a single oral administration of 0.3, 0.6 and 1.2 mg/kg of codeine and a single intravenous administration of 0.2 mg/kg morphine (positive control). This was repeated until all horses received all treatments. The order of treatment for each horse randomly assigned using a computerized random number generator with a minimum washout period of 2-weeks observed between treatments. The 0.6 mg/kg codeine dose was selected based on morphine and M6G concentrations achieved in a previously published study [14]. The 0.3 and 1.2 mg/kg doses were determined by halving and doubling the previously studied dose. Prior to drug administration, a 14-gauge catheter was placed, using aseptic technique, in one external jugular vein for sample collection. Horses that received morphine had a second catheter placed (in the contralateral jugular vein) for drug administration.

Horses were fasted for 12 hours prior and 2 hours post drug administration. Codeine sulfate tablets (Lannett, Philadelphia, PA) were dissolved in water and delivered via a dosing syringe directly into the caudal portion of the oral cavity. Saline was administered in a similar manner. Morphine (Hospira, Lake Forest, IL) was administered via an intravenous jugular vein catheter. Following drug administration, the catheter was flushed with 10 ml of a dilute heparinized saline solution (10 IU/mL). The dosing catheter was removed following dosing.

### Sample collection

Blood samples for drug and metabolite concentration determination were collected at time 0 (prior to drug administration) and at 5, 10, 15, 30, and 45 minutes, and 1, 2, 3, 4, 5, 6, 8, 12, 18, 24, 36, 48, 72 and 96 hours post administration of codeine and morphine from the jugular vein catheter. Catheters were removed following collection of the 24-hour sample with the remaining samples collected by direct venipuncture. Blood samples were collected into EDTA blood tubes (Kendall/Tyco Healthcare, Mansfield, MA), centrifuged at 3000 x g and plasma immediately transferred into storage cryovials and stored at − 20^°^C until analysis.

### Analysis of blood samples

Plasma samples were analyzed for determination of codeine, morphine and metabolite concentrations using liquid chromatography tandem mass-spectrometry (LC-MS/MS) and previously validated and published methods [14, 16]**.**

### Pharmacokinetic analysis

Pharmacokinetic analyses for parent compounds and metabolites were conducted using a commercially available pharmacokinetic software program (Phoenix Winnonlin v8.2, Pharsight, Princeton, NJ) and non-compartmental analysis. The slope of the terminal portion of the curve, lambda *z* (λ_*z*_) was used to calculate half-life (HL λ_z_) using the eq. 0.693/ λ_z_. The area under curve (AUC) from time 0 to infinity (AUC_0 → ∞_) was obtained by using the linear up log down trapezoidal rule, then dividing the last plasma concentration by the terminal slope extrapolated to infinity. Clearance (Cl) and the apparent volume of distribution at steady state (V_ss_) were determined for morphine by the pharmacokinetic software using the following formulas:

$$\mathrm{Cl}=\mathrm{Dose}/{\mathrm{AUC}}_{0\to \propto }$$

$${V}_{ss}={MRT}_{\propto }\ x\ Cl$$

where MRT is the mean residence time.

### Physiological responses and behavioral monitoring

Prior to commencement of the study, horses were equipped with two Step Monitors (SAM3, Seattle, WA) programmed to count the number of steps taken each minute as described previously [17]**.** The number of steps taken by the horses’ instrumented front and hind limbs were recorded for a minimum of 30 minutes prior to and for 6 hours post drug or saline administration. Horses were also equipped with a Holter monitor (Forrest Medical, East Syracuse, NY) and the heart rate recorded continuously for a minimum of 30 minutes pre- and 6 hours post drug administration.

Gastrointestinal borborygmi were determined by auscultation of each abdominal quadrant for 30 seconds and a numerical scoring system ranging from 0 to 4 as described previously [37]**.** Auscultation was performed prior to drug administration and at 30 and 45 minutes and 1, 2, 4, 6 and 24 hours post drug administration in all horses. Defecation incidence as well as the number of fecal piles, number of fecal balls within a pile and consistency (normal, wet or dry) was recorded throughout the sampling period. Fecal piles were removed from the stall at each time point. Pile size was considered small if less than 15 fecal balls, average if 15–30 and large if greater than 30.

Other notable physiologic and behavioral observations were recorded throughout.

### Assessment of thermal nociception

A commercially available wireless device (Topcat Metrology, UK) was used, as described previously [36, 38–41], for thermal threshold testing. Briefly, prior to the commencement of the study, an area on the outside of the metacarpus was shaved for application of the temperature probe. For assessment of thermal thresholds, the temperature probe was placed in direct contact with the skin and the nylon strap tightened around the leg and proper and consistent contact ensured as previously described [37]**.** Skin temperature at the contact point of the thermal element and the ambient temperature was recorded prior to each reading. An infrared remote, located outside the stall, was used to adjust the temperature of the thermal element at a rate of 1.1 °C per second. The temperature at which the horse responded to the stimulus (stomping, lifting, pawing or touching their nose to the right front leg) or when the probe reached 55 °C (automatic shut-off to avoid burns) was recorded as the threshold temperature for the time point. Baseline thermal measurements were taken in triplicate the morning of drug administration (approximately 30–60 minutes prior to drug administration) with a 5-minute interval between each reading. Thermal assessments were also conducted at 15, 30, 45, 60, 90, 120, 180, 240 and 360 minutes after administration of codeine, saline, or morphine.

### Statistical analysis

Thermal nociceptive thresholds were standardized to %TE for comparability, as described previously [36], using the formula:

$$\% TE=100\ x\ \left[\left({T}_t-{T}_0\right)/\left({T}_C-{T}_0\right)\right]$$

where T_T_ represents the thermal threshold, T_0_ the skin temperature and T_C_ the thermal nociceptive cut-off temperature.

Statistical analyses to assess significant differences in pharmacodynamic parameters between baseline and each time point were conducted using commercially available software (Stata/IC 13.1, StataCorp LP, TX, USA). Data were analyzed using a paired t-test and a significance level of 0.05.

## Supplementary Information

**Additional file 1.**

## Data Availability

The datasets used and/or analyzed during the current study are available from the corresponding author on reasonable request.

## Abbreviations

AUC: Area under the curve
CBC: Complete blood count
Cl: Clearance
C_max_: Maximum concentration
C6G: Codeine-6-glucuronide
LC-MS/MS: Liquid chromatography mass spectrometry/mass spectrometry
LOQ: Limit of quantitation
M3G: Morphine-3-glucuronide
M6G: Morphine-6-glucuronide
MRT: Mean residence time
%TE: Thermal Exclusion
T_max_: Time of maximum concentration
T_T_: Thermal Threshold
T_0_: Skin Temperature
T_C_: Thermal nociceptive cut-off temperature
V_ss_: Volume of distribution at steady state
