# Supplementary material for: Pharmacokinetics, adverse effects and effects on thermal nociception following administration of three doses of codeine to horses
Source: BMC Vet Res. 2022 May 25;18:196. doi: 10.1186/s12917-022-03299-0 (PMC9131543; doi:10.1186/s12917-022-03299-0)
Supplement: Supplementary file 1 — Additional file 1. [file 12917_2022_3299_MOESM1_ESM.docx]

**Supplementary Table 1**. Accuracy and precision values for LC-MS/MS analysis of codeine, morphine, morphine 3-glucuronide (M3G), morphine 6-glucuronide (M6G) and norcodeine in plasma.

| Analyte | Concentration  (ng/mL) | Intra-day accuracy (% nominal concentration) | Intra-day precision  (% relative SD) | Inter-day accuracy (% nominal concentration) | Inter-day precision  (% relative SD) |
| --- | --- | --- | --- | --- | --- |
| Codeine |  |  |  |  |  |
|  | 0.75 ng/mL | 107 | 3.0 | 104 | 4.0 |
|  | 40 ng/mL | 108 | 3.0 | 107 | 3.0 |
|  | 400 ng/mL | 106 | 8.0 | 106 | 4.0 |
| Morphine |  |  |  |  |  |
|  | 0.75 ng/mL | 111 | 7.0 | 108 | 8.0 |
|  | 40 ng/mL | 104 | 3.0 | 104 | 3.0 |
|  | 400 ng/mL | 105 | 6.0 | 105 | 3.0 |
| M6G |  |  |  |  |  |
|  | 0.75 ng/mL | 110 | 6.0 | 103 | 7.0 |
|  | 40 ng/mL | 105 | 4.0 | 107 | 3.0 |
|  | 400 ng/mL | 111 | 6.0 | 111 | 3.0 |
| M3G |  |  |  |  |  |
|  | 0.75 ng/mL | 100 | 7.0 | 95.0 | 5.0 |
|  | 40 ng/mL | 103 | 5.0 | 108 | 6.0 |
|  | 400 ng/mL | 106 | 6.0 | 109 | 3.0 |
| C6G |  |  |  |  |  |
|  | 0.75 ng/mL | 101 | 6.0 | 98.0 | 5.0 |
|  | 40 ng/mL | 100 | 3.0 | 100 | 2.0 |
|  | 400 ng/mL | 115 | 8.0 | 102 | 4.0 |
| Norcodeine |  |  |  |  |  |
|  | 0.75 ng/mL | 94.0 | 7.0 | 95.0 | 5.0 |
|  | 40 ng/mL | 98.0 | 2.0 | 97.0 | 2.0 |
|  | 400 ng/mL | 112 | 8.0 | 107 | 4.0 |
